# Study protocol for a randomised cross-over trial of *N*eurally adjusted ventilatory *A*ssist for *N*eonates with *C*ongenital diaphragmatic hernias: the NAN-C study

**DOI:** 10.1186/s13063-023-07874-0

**Published:** 2024-01-20

**Authors:** Grace Poole, Christopher Harris, Sandeep Shetty, Theodore Dassios, Allan Jenkinson, Anne Greenough

**Affiliations:** 1 Neonatal Intensive Care Unit, King’s College Hospital NHS Foundation Trust, London, UK; 2 Neonatal Intensive Care Unit, St. George’s University NHS Foundation Trust, London, UK

**Keywords:** Neurally adjusted ventilatory assist, NAVA, Congenital diaphragmatic hernia, CDH, Neonatal intensive care, Neonatology

## Abstract

**Background:**

Neurally adjusted ventilatory assist (NAVA) is a mode of mechanical ventilation that delivers oxygen pressures in proportion to electrical signals of the diaphragm. The proportional assistance can be adjusted by the clinician to reduce the patient’s work of breathing. Several case series of infants with congenital diaphragmatic hernias (CDH) have shown that NAVA may reduce oxygenation index and mean airway pressures. To date, no clinical trial has compared NAVA to standard methods of mechanical ventilation for babies with CDH.

**Methods:**

The aim of this dual-centre randomised cross-over trial is to compare post-operative NAVA with assist control ventilation (ACV) for infants with CDH. If eligible, infants will be enrolled for a ventilatory support tolerance trial (VSTT) to assess their suitability for randomisation. If clinically stable during the VSTT, infants will be randomised to receive either NAVA or ACV first in a 1:1 ratio for a 4-h period. The oxygenation index, respiratory severity score and cumulative sedative medication use will be measured.

**Discussion:**

Retrospective studies comparing NAVA to ACV in neonates with congenital diaphragmatic hernia have shown the ventilatory mode may improve respiratory parameters and benefit neonates. To our knowledge, this is the first prospective cross-over trial comparing NAVA to ACV.

**Trial registration:**

NAN-C was prospectively registered on ClinicalTrials.govNCT05839340 Registered on May 2023

**Supplementary Information:**

The online version contains supplementary material available at 10.1186/s13063-023-07874-0.

## Administration information

Note the numbers depicted in braces throughout the protocol refer to *SPIRIT* checklist item number [1]. The order of the items has been modified to group similar items.

**Table:** 

Title {1}	Study protocol for a randomised cross-over trial of *N*eurally adjusted ventilatory *A*ssist for *N*eonates with *C*ongenital diaphragmatic hernias: the NAN-C study
Trial Registration {2a and 2b}	ClinicalTrials.gov. NCT05839340. https://beta.clinicaltrials.gov/search?term=NCT05839340 The protocol is registered on clinicaltrials.gov. All modifications to the original protocol are shown on clinicaltrials.gov. All items from the World Health Organisation Trial Registration Data Set are included in the trial registry.
Protocol Version {3}	NAN-C Protocol Version 11.0 – 12th May 2023
Funding {4}	This study is being funded by King’s College London and St. George’s University Hospital NHS Foundation Trust Neonatal Intensive Care Unit. The funding sources do not have any role in the study design, analysis, interpretation, or publication of the manuscript.
Author Details {5a}	Professor Anne Greenough. Professor of Neonatology and Clinical Respiratory Physiology. Department of Women and Children’s Health, School of Life Course sciences. Faculty of Life Sciences and Medicine, King’s College London, United Kingdom. anne.greenough@kcl.ac.uk Dr. Christopher Harris. PHD. Consultant Neonatologist. King’s College Hospital NHS Foundation Trust, London, United Kingdom. christopher.harris@kcl.ac.uk. Dr. Sandeep Shetty. Consultant Neonatologist, St. George’s University Hospital NHS Foundation Trust, London, United Kingdom. sandeep.shetty@stgeorges.nhs.uk. Dr. Grace Poole. Academic Foundation Doctor, King’s College Hospital NHS Foundation Trust, London, United Kingdom. grace.poole5@nhs.net
Study Sponsor {5b and 5c}	Jasmine Palmer, Research & Innovation Governance Manager, King’s College Hospital NHS Foundation Trust. jasmine.palmer1@nhs.net Sam Hollingworth, Research Governance and Facilitation Officer, St. George’s University Hospital NHS Foundation Trust. sahollin@sgul.ac.uk.
Role of Sponsor {5c}	The study sponsor has no involvement in study design, data collection, management, analysis or interpretation, report writing or the decision to submit the study for publication.

## Introduction

### Background and rationale {6a}

Congenital diaphragmatic hernia (CDH) occurs due to an incomplete fusion of the diaphragm during foetal development, enabling abdominal viscera to herniate into the thoracic cavity [2]. Herniation may disrupt development of the lung and associated vasculature, resulting in pulmonary hypoplasia and pulmonary hypertension [3]. Neonates typically undergo surgical reduction in the first few days after birth [4]. The ventilation-perfusion mismatch often seen in infants with CDH can make the post-operative ventilation of this population challenging. The use of mechanical ventilation (MV) is the standard care for babies with CDH in neonatal intensive care units (NICU) across the UK [5]. The use of MV in this patient group, however, can injure the hypoplastic and contralateral lung, termed ventilator-associated lung injury (VALI) [6]. The optimal mode of ventilation to prevent VALI in neonates with CDH remains unclear [4].

One method hypothesised to confer protection to the CDH hypoplastic lung is neurally adjusted ventilatory assist (NAVA). When ventilating with NAVA, a nasogastric tube is passed that contains miniaturised electrodes to detect electrical diaphragmatic signals (Edi). These signals trigger the ventilator to deliver positive pressure at a level, set by the clinician, at the start of inspiration. Inflations are off cycled when the Edi falls by a pre-specified amount [7]. Due to this, NAVA may trigger ventilatory support earlier in the respiratory cycle compared to pressure-triggered ventilatory methods (PTV), where the infant must initiate a sufficient change in pressure or flow to trigger ventilatory support [8]. As a consequence, several small studies have demonstrated that NAVA improves patient-ventilatory asynchrony due to reduced trigger delays and auto or double triggering [9].

In the CDH population, it has been hypothesised that a structurally abnormal diaphragm may impede Edi signal detection and negate the benefits of NAVA. In a retrospective 1:2 matched case-control study, however, there was no significant difference in the Edi signal between infants with CDH ventilated with NAVA and those without [10]. In a retrospective cohort study, five out of seven infants that underwent a surgical patch repair for their CDH had active Edi signals [9]. Several small studies have suggested NAVA is superior compared to assist control ventilation. A retrospective cohort analysis of 15 CDH neonates supported with 72 h of NAVA showed reductions in the peak inspiratory pressure (PIP), mean-airway pressure (MAP) and resulted in less sedative-medication use [11]. Another retrospective cohort of 12 infants in a single centre showed improvements in oxygenation index (OI) on NAVA, compared to pressure-support modes [12].

While results from retrospective analysis have been promising, to our knowledge there has been no prospective crossover trial investigating NAVA for infants with CDH.

### Objectives {7}

Our objective is to determine if for neonates with CDH, NAVA will result in a better OI, compared to ACV. The secondary objective is to determine other clinically important outcomes including sedative medication use.

## Methods

### Trial design {8}

NAN-C is a dual-centre, randomised, open-label cross-over trial designed with a superiority framework. Neither the clinical team nor study investigators can be blinded to the study intervention, but the study statistician will be blinded to each study arm.

### Study setting {9}

This dual-centre randomised cross-over trial will involve two NICUs in the UK. NAN-C was granted a favourable ethical opinion by the West of Scotland Research Ethics Committee (REC).

### Eligibility criteria {10}

We will include neonates (< 28 days of life) with CDH. Eligible Infants will be identified daily by a researcher following discussion with the clinical team. The full inclusion and exclusion criteria can be found outlined in Table 1. Parents of participants meeting all the screening inclusion criteria and none of the screening exclusion criteria will be approached to give consent for their infant’s participation. Post-operatively, infants will then be enrolled within the study and proceed to a ventilatory support tolerance trial (VSTT).

**Table 1:** Inclusion and exclusion criteria for participation {10}

Inclusion criteria	Exclusion criteria
▪ Infants born with a congenital diaphragmatic hernia at a gestation age of more than 34 weeks	▪ Known chromosomal anomaly
	▪ Cardiac anomalies expected to require corrective surgery in the first 60 days of birth
	▪ Renal anomalies associated with oligohydramnios
	▪ Skeletal deformities impeding lung or thoracic development
	▪ Severe anomalies of the central nervous system. Including neurological trauma or raised intracranial pressure
	▪ Neuromuscular blocking agent use
	▪ Contraindication to nasogastric tube insertion
	▪ Nitric oxide requirement
	▪ FiO_2_ greater than 80% at the end of the ventilatory support tolerance trial

For the VSTT, neonates will be stabilised for an hour on ACV on the servo-n ventilator (Maquet Critical Care, Solna, Sweden). The positive end-expiratory pressure (PEEP) will be kept 4–5cm H_2_O and inflation time at 0.36–0.4 s. The fraction of inspired oxygen concentration (FiO_2_) will be adjusted with the aim of maintaining oxygen saturations between 85 and 95%. At this stage, infants requiring an FiO_2_ greater than 80% to maintain their oxygen saturation, or requiring nitric oxide, will be excluded.

### Sample size {14}

The primary outcome is OI. To detect a minimum clinically important difference of one standard deviation with 80% power and 5% significance eighteen infants will be recruited for the trial.

### Recruitment {15}

Foetuses with CDH are usually identified via antenatal screening and clinical NICU teams alerted prior to their delivery. Infants with CDH, who may be suitable for the clinical trial, will be identified following a daily discussion with the NICU team. Following delivery, infants will be screened for their eligibility to participate within the trial. As part of the routine standard of care, CDH infants will receive an echocardiogram within the first few days of life, enabling the identification of any associated cardiac anomalies.

On average, each NICU admits one CDH neonate per month. We aspire to recruit eighteen infants between August 2023 and August 2024.

### Consent or assent {26a and 26b}

Parents/legal guardians of infants with CDH that meet eligibility criteria will be informed of the trial by a named clinical consultant. At this stage, the clinical team will provide consenting adults with a participant information sheet (PIS). If interested in participating, a member of the research team will organise a face-to-face interview with parents/legal guardians. This meeting will provide parents an opportunity to ask any further questions regarding the study. It will be emphasised in this meeting that choosing not to participate will not affect their infant’s care. Informed written consent will be obtained.

No biological specimens will be collected or stored for study purposes. Blood tests to monitor the effect of ventilatory settings will be performed as part of routine clinical care.

### Allocation and blinding {16a, 16b, 16c, 17a and 17b}

Each infant in this study will be randomised to receive NAVA or ACV in a 1:1 ratio using computer generation sequence methodology.

The clinical team and study investigators will know which study intervention is assigned to each patient. The independent statistician will be blinded to each study arm.

### Provisions for post-trial care {30}

Post-trial care is the responsibility of treating clinicians. Patients will not receive financial compensation for their participation in the study.

### Participant timeline {13}

**Fig. 1:**
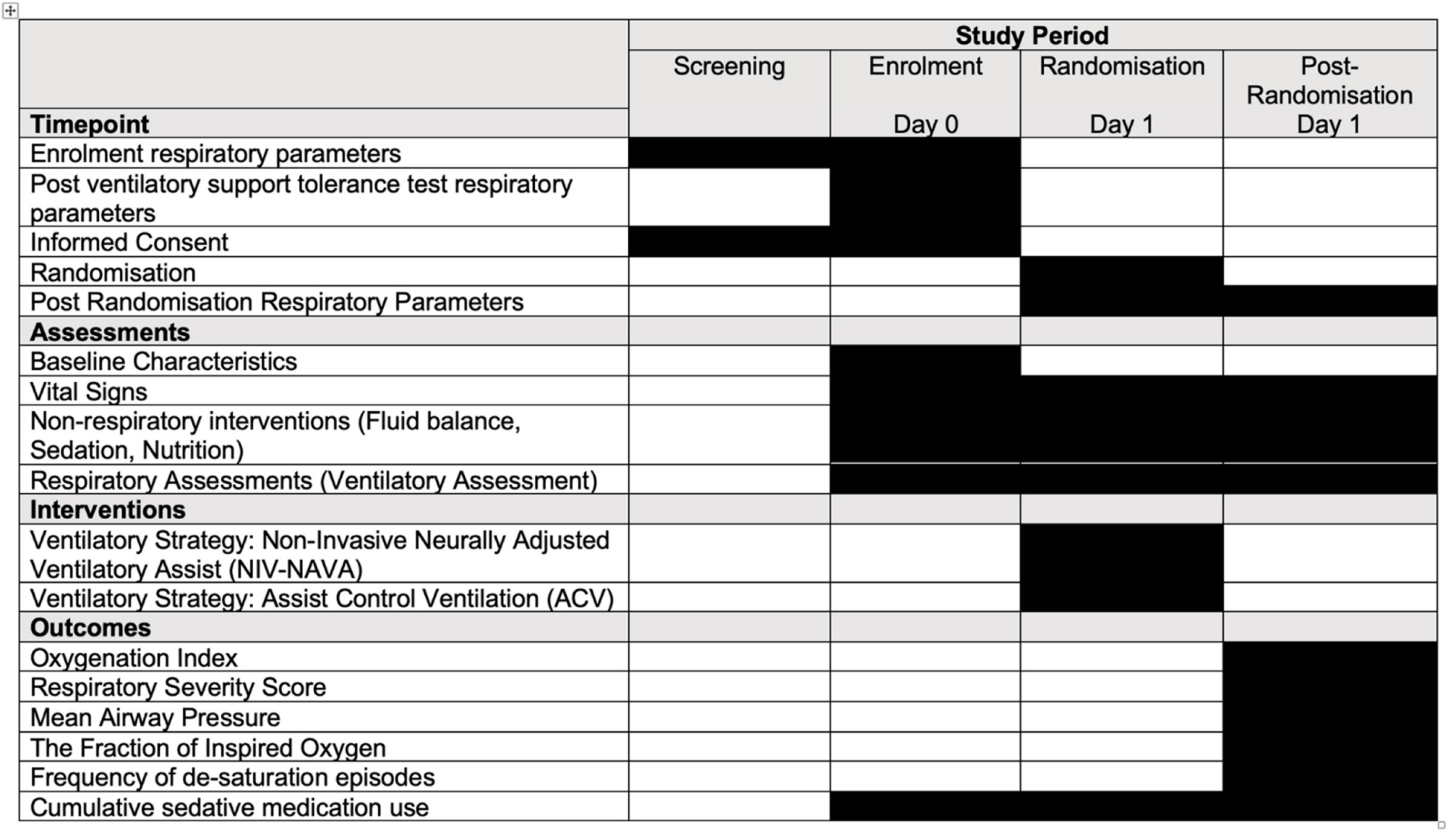
Participant timeline from screening to discharge from the NAN-C trial

Screening of participants begins once they have received 24 h of invasive ventilation. Eligible patients will be enrolled into a VSTT. If participants pass the VSTT, they will be randomised to receive either NAVA or ACV first. Participants remain on the assigned ventilator mode for 4 h. Primary outcomes will be recorded for the final 30 min of each ventilation period. Infants will undergo a 20-min stabilisation period prior to receiving the second mode of ventilation for 4 h. There are no follow-up visits of data collection following the total ventilation period (Fig. 1).

### Research ethics and approval {24}

Favourable ethical opinion was granted by the West of Scotland REC. Written, informed consent to participate will be obtained from all parents/legal guardians of eligible children.

### Plans for communicating important protocol amendments to relevant parties (e.g. trial participants, ethical committees) {25}

The co-ordinating committee is responsible for the distribution of protocol amendments to site investigators. Site investigators are responsible for the distribution of amendments to all staff involved within the study and for obtaining approval from the local research ethics committee as required.

### Authorship {31b}

Investigators will be invited to contribute to writing and reviewing the primary manuscripts and abstracts.

### Declaration of interests {28}

The authors declare no conflicts of interest.

## Oversight and monitoring

### Composition of the co-ordinating centre and trial steering committee {5d}

The research and innovation (R&I) department at King’s College Hospital NHS Foundation Trust will oversee all contracts. King’s College London, UK, will oversee insurance and financial arrangements. The executive steering committee (EC) will consist of the lead investigators (AG, CH, SS) that are experienced neonatal intensive care consultants, with support of the study statistician (TD). The EC will oversee all aspects of the study including implementation and daily operations. The EC will meet monthly during the trial. NICU teams will retain clinical responsibility for the neonate throughout the trial’s duration. Members of the research team (GP and AJ) will be responsible for primary data collection. The organisational team will meet on a weekly basis to discuss any issues pertaining to data collection.

### Composition of the data monitoring committee, it’s role in reporting and structure {21a}

The chief investigator (CH) will act in an advisory capacity to safeguard the interests of participants and assess the safety of interventions and monitor trial conduct. The R&I department will have the ability to request additional safety analyses and make recommendations about the safe conduct of the trial.

### Adverse event reporting and harms {22}

Although we anticipate no additional risks incurred by infants participating in the study, the following monitoring approach will be employed to ensure the safety of all patients. All serious adverse events (SAE) will be reported to a legal representative, manufacturer, sponsor and R&I within one working day of the investigator becoming aware. Related and unexpected SAEs will be reported within 15 working days. All SAEs will be recorded on an SAE form by the chief investigator (CH).

The manufacturer has a legal obligation to report all events that need to be reported to the Nominated Competent Authority after a link is established between the event and device: 2 days following the awareness of the event for Serious Public Health Threat, 5 days following awareness of the event for Death or unanticipated serious deterioration in health and 30 days following the awareness of the event for all other events meeting the SAE criteria.

Where the event is unexpected and thought to be related to the intervention/treatment/procedure this must be reported by the Investigator to the REC and Health Research Authority, using the SAE Report form for non-CTIMPs (available from the HRA website) within 15 days.

### Frequency and procedures for auditing trial conduct {23}

On-site and virtual monitoring activities will be used to ensure the quality of data captured, study operations and patient safety. A risk-based approach will govern the monitoring activities of each NICU. The site investigator will permit study-related monitoring, audits, and inspections by the REC, the sponsor, of all study-related documents. The site investigator will ensure the capability for inspections of applicable study-related facilities.

## Interventions

### Intervention description {11a}

A six-French (6 FR) specialised nasogastric tube (EAdi Catheter) is inserted to monitor electromyogram of the crural diaphragm. Correct position of the EAdi catheter is confirmed as per instructions of the manufacturer (Maquet, Servo-n user manual version 4.1) [13]. The guide function displays the retrocardiac echocardiograph. Correct positioning is when the P waves and QRS complexes are visible in the upmost leads and then decrease in size until the P waves disappear in the lowest leads. Coloured highlighting of the central two leads will also appear once the catheter is in the correct place. Once correct positioning is confirmed, the catheter will be securely attached to the infant’s face using an adhesive dressing.

Before infants are changed to NAVA mode (*Servo-n ventilator, Maquet Critical Care, Solna, Sweden*), the NAVA level will be adjusted by the clinician, so the display pressure waveform closely matches the actual pressure waveform, aiming for peak Edi between 5 and 15 microvolts (μV).

On recruitment into the trial, infants will undergo a 1-h VSTT. At King’s College Hospital NHS Foundation Trust infants will be transferred from the SLE 6000 Ventilator (Software versions 4.3; SLE Ltd; South Croydon, UK) to the servo-n ventilator (Maquet Critical Care, Solna, Sweden) for the VSTT. At St. George’s University Hospital NHS Foundation Trust, infants will remain on ACV using the servo-n ventilator (Maquet Critical Care, Solna, Sweden). At this stage, infants requiring an FIO2 of more than 80% to maintain SpO2 85–95%, or requiring nitric oxide infusion, will be excluded.

**Fig. 2:**
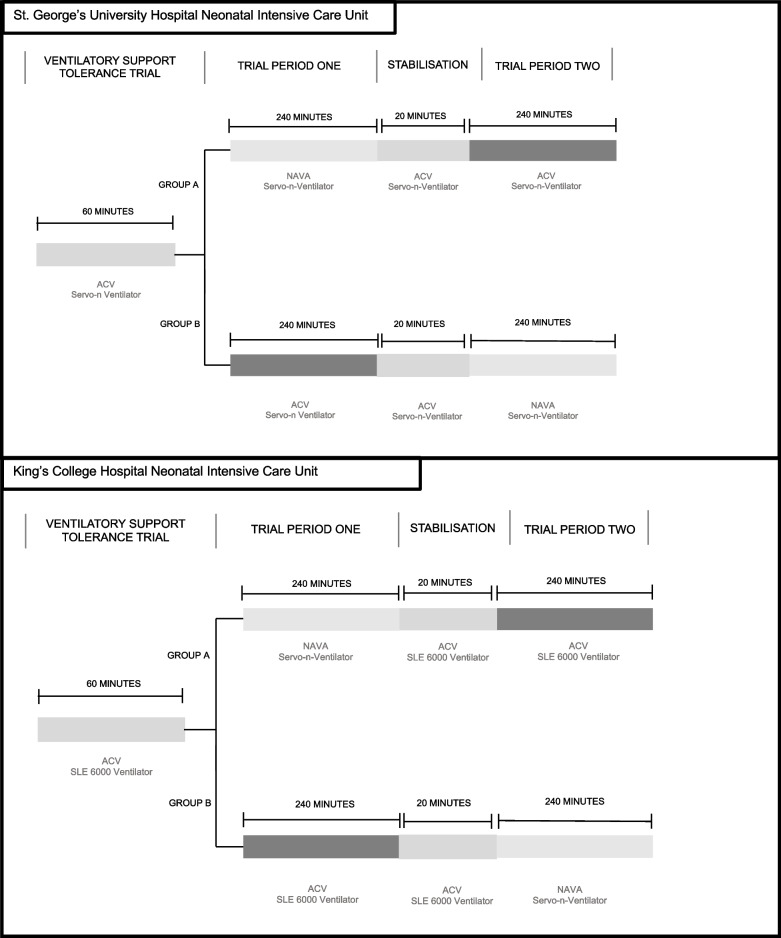
Ventilation protocol for the NAN-C trial. At King’s College Hospital NHS Foundation Trust (KCH), infants are routinely ventilated using assist control ventilation on the SLE 6000 ventilator. If infants meet the eligibility criteria, they will be transferred from ACV on the SLE 6000 to ACV on the servo-n ventilator (in the above diagram, ventilation machine changes depicted by arrows). Infants will be stabilised on the servo-n ventilator for 1 h in the ventilatory support tolerance trial. If suitable, infants will then be randomised to receive either ACV or neutrally adjusted ventilator assist (NAVA) for 4 h. Following a 20-min stabilisation period, they will then receive the second mode of ventilation. At St. George’s University Hospital (SGH), infants are routinely ventilated on ACV on the servo-n ventilator. Following the VSTT, they will be randomised to either NAVA or ACV on the servo-n ventilator

Infants will then be randomised to receive either ACV or NAVA ventilation modes on the servo-n ventilator for the first 4-h period. All infants will then undergo a 20-min stabilisation period. Following the stabilisation period, infants will then cross-over to receive the alternative ventilatory mode on the servo-n ventilator (Fig. 2)*.*

### Criteria for discontinuing or modifying allocated interventions {11b}

The back-up mode set is pressure control ventilation (PCV). The FiO_2_ will be adjusted with the aim of maintaining oxygen saturations between 85 and 95, as per recommendations of the CDH Euro Consortium [4]. The trial will be ceased in any infant with an increase in fraction of inspired oxygen concentration more than 30%. Any infant failing to trigger the ventilator will be excluded. In all cases, reasons for exclusion will be thoroughly documented.

### Strategies to improve adherence to interventions {11c}

To improve adherence to study interventions, a researcher will remain by the bedside for the study period recording any changes made by the clinician responsible for adjustment of the ventilator, including any deviations from protocol.

### Relevant concomitant care permitted or prohibited during the trial {11d}

All non-respiratory NICU care will be at the discretion of the responsible clinical consultant and in line with the NICU’s guidelines. Guidelines for the administration of sedative medication and analgesia are provided by NICU, with a recommendation to use the lowest possible dose of sedative medication as required. We will record the daily dose of analgesia, sedative, and neuroleptic medications. All infants will be reviewed by a dietician to ensure the infant is receiving adequate calories, protein, and nutrients to meet their energy requirements.

### Explanation for choice of comparators {6b}

NAVA is a unique, closed-loop mode of mechanical ventilation that has been shown to optimise patient-ventilatory synchrony by responding directly to neonatal diaphragmatic signalling [9]. The aim of this study is to determine if NAVA is superior to ACV in achieving clinically important outcomes. ACV was chosen as the comparator as it is the standard of care for ventilating infants in many NICUs.

### Outcomes {12}

The primary outcome of the study is the average OI for the final 30 min of each 4-h ventilatory period. OI is calculated as MAP x FiO_2_ × 100/PaO_2_ [14]. The OI is often used as an indicator of hypoxemic respiratory failure (HRF) and in infants with CDH is the leading criterion to administer inhaled nitric oxide (iNO) or initiate extracorporeal membrane oxygenation therapy (ECMO) [15, 16]. Previous research has demonstrated a 1-h period is sufficient to compare OI between NAVA and ACV [17]. We have decided on a 4-h period to allow neonates sufficient time to stabilise on the ventilatory mode.

The secondary outcome measures and monitored variables will include:

(i) The average respiratory severity score (RSS) during the final 30 min of each 4-h ventilation period. The RSS is calculated as FIO_2_ x MAP. It will be calculated prior to randomisation, post randomisation and following cross-over.
(ii) Average MAP during the final 30 min of each 4-h ventilation period.
(iii) Initial ventilator settings and the number of de-saturations, defined as an SpO2 of less than 88%, will be noted for each neonate.
(iv) Average PIP during the final 30 min of each 4-h ventilation period.
(v) Cumulative total concentration of morphine-equivalent use will be recorded 4-h prior to ventilation with ACV or NAVA. In addition, total morphine-equivalent use during each 4-h ventilation period will be recorded and compared.
(vi) Cumulative total concentration of midazolam use will be recorded 4-h prior to ventilation with ACV or NAVA. In addition, midazolam use during each 4-h ventilation period will be recorded and compared.

### Dissemination policy {31a and 31c}

We plan to submit a manuscript for publication in an international journal and submit an abstract for presentation at an international meeting. For 6 months following the publication of the final study results, investigators will be granted priority for secondary data analyses. Following this period, requests for de-identified data will be considered. Requests for data presented in the final paper should be submitted via email to Professor Anne Greenough.

## Data collection and management

### Data collection methods {18a}

As per standard of care, outcome, baseline, and clinical care data are recorded by the clinical NICU team in the patient’s notes, which will serve as documents for trial data. Additional information related to trial outcomes will be recorded by authorised and trained members of the research team.

### Plans to promote participant retention and complete follow-up {18b}

As infants are receiving invasive MV for the duration of the trial, we expect complete follow-up in 95% of randomised patients. We anticipate a 5% loss-to-follow-up rate comprised of infants transferred to other hospitals, consent is withdrawn or become too unwell to participate in the trial.

### Data management {19, 27, 29}

Data collected during the trial will be handled and stored in accordance with the General Data Protection Regulation and Data Protection Act (2018) that requires records to be de-anonymised as soon as practical to do so.

At individual sites, data will be downloaded from ventilators using a secure password-encrypted USB device and uploaded on password-protected servers and anonymised at the point of entry. Anonymised data will be identified by a unique trial identification number. A trial enrolment log at the sites will list all identification numbers.

Original paper study documents will be retained at trial sites in a secure location until the children are 25 years old, as per protocol.

Anonymised data will be shared with other researchers to aid further study at the discretion of the principal investigator, with permission from the parent/legal guardian.

### Statistical methods {20a}

Data will be analysed following intention-to-treat. Descriptive statistics will be used to describe baseline data including demographics of both treatment groups. Means, medians, ranges and confidence intervals, proportions and percentages will be used as appropriate.

Primary and secondary outcome data will be assessed for normality. Non-parametric

Wilcoxon rank sum will be used to analyse non-normally distributed data. Where data is normally distributed, a *t*-test will be used to compare study arms. 95% confidence intervals will be reported throughout.

### Methods for additional analyses (Subgroup Analyses) {20b}

We have planned a subgroup analysis based on neonates that underwent FETO. A second analysis of outcomes will be done with adjustment for covariates (baseline clinical variables specified a priori with potential confounding effects on MV).

### Methods in analysis to handle protocol non-adherence and statistical methods to handle missing data {20c}

Data will be analysed according to intention-to-treat principles. A descriptive analysis on participants who have withdrawn from the study due to clinical instability or withdrawal of consent will be recorded.

### Interim analysis {21b}

We will conduct an interim analysis when nine infants have been recruited. An interim analysis has been planned as previous research investigating NAVA, compared to ACV, for infants with bronchopulmonary dysplasia demonstrated significantly better OI on the former mode of ventilation [16]. To preserve the type I error at 5%, the interim analysis will be conducted at 0.01 with the final analysis conducted using 0.04. This will give an overall type 1 error rate (*significance level*) of 5% [(1–0.01) × (1–0.04) = 0.95 = 1–0.05]. If the interim analysis shows *p* < 0.05, then the trial will stop, and the final analyses will be conducted using patients treated up until that point.

### Plans for collection, laboratory evaluation, and storage of biological specimens for genetic or molecular analysis in this trial/future use {33}

No biological specimens will be collected or retained for this trial or future use.

### Patient and public involvement

Parents of infants with CDH in the NICU were consulted regarding the trial design, consent process and parent-facing materials. Members of the public comprising the REC commented on the trial protocol.

## Discussion

Multiple retrospective observational studies comparing NAVA to ACV suggest NAVA may benefit infants with CDH [11, 18–21]. To our knowledge, there has been no prospective trial comparing NAVA to ACV. In this study protocol, we describe a dual-centre, sufficiently powered, randomised cross-over trial needed to determine the most appropriate method of ventilation for infants with CDH.

In the following section, we discuss the rationale for our primary outcome, the definition of the primary outcome, standardisation of the method and other unique aspects of this trial protocol.

Regarding the primary outcome, OI is calculated as MAP × FiO_2_ × 100/PaO_2_ [14]. OI is commonly used to assess the severity of HRF and PPHN of the new-born in NICU. OI is considered a better index than PaO_2_/FiO_2_ ratio as it includes MAP, an important determinant of oxygenation [22]. In infants with CDH, OI is the leading criterion to administer iNO or initiate ECMO [15, 16]. There are several disadvantages to the use of OI in NICU. It requires an indwelling arterial line to obtain a blood gas sample, the sample is dictated by the location of the arterial line and OI can only be intermittently measured when blood gases are taken. We did consider the use of oxygen saturation index (OSI = MAP × FiO_2_ x 100: SpO_2_), which has been shown as a good correlate to OI for infants with HRF [15, 16, 23]. However, due to only one retrospective cohort study of 33 infants with CDH investigating OSI as a correlate for OI, we have decided to utilise OI during this study [16]. NAN-C may be criticised due to a lack of clinical outcomes. Due to a lack of prospective trials investigating OI, MAP and RSS for infants with CDH ventilated with NAVA, we hope our cross-over trial will add to the literature and form a basis for future clinical correlation.

A criticism of previous literature investigating ventilation modes for infants with CDH is the small sample size and single-centre nature of research reflecting the incidence of CDH. Strengths of the study include dual-centre participation.

Lack of familiarity with NAVA may be a study limitation. However, at both centres training will be provided on ventilator modes, and a VSTT is mandatory prior to randomising patients. The mandatory VSTT also safeguards the fidelity of the trial, by ensuring centres can deliver the treatments and record the data according to the proposed methods.

Several challenging aspects were encountered in designing the trial. The greatest challenge involves one trial site (King’s College Hospital, London) routinely using SLE 6000 ventilator machines that are unable to deliver NAVA. Given this, infants at this trial site must be transferred from the SLE 6000 ventilator to servo-n-ventilator machines. A change in ventilator machines introduces a theoretical risk of extubation and represents a necessary deviation in protocol between trial sites. The resultant version 11.0 of the NAN-C Study Protocol reflects a study designed to accurately capture the populations of interest, enrol, and randomise them to well-defined ventilation algorithms, minimise bias and analyse results in a comprehensive manner.

In conclusion, we describe a protocol for a dual-centre randomised cross-over trial in infants with CDH to compare OI on NAVA mode, compared to ACV. We hope our study may inspire further research investigating the clinical outcomes of NAVA for infants with CDH.

## Trial status

This publication is based on version 11.0 of the NAN-C study protocol (12th May 2023). Recruitment is scheduled to begin on 12 June 2023, with two sites. We anticipate completing the study in June 2024.

### Supplementary Information

**Additional file 1.**

## Abbreviations

ACV: Assist control ventilation
CDH: Congenital diaphragmatic hernia
ECMO: Extracorporeal membrane oxygenation
Edi: Electrical diaphragmatic signals
FiO_2_: Fraction of inspired oxygen concentration
HRF: Hypoxemic respiratory failure
iNO: Inhaled nitric oxide
MAP: Mean airway pressure
MV: Mechanical ventilation
NAVA: Neurally adjusted ventilatory assist
NICU: Neonatal intensive care unit
OI: Oxygenation index
OSI: Oxygen saturation index
PaO2: Partial pressure of oxygen
PCV: Pressure control ventilation
PEEP: Positive end-expiratory pressure
PIP: Peak inspiratory pressure
PIS: Participant information sheet
PPHN: Pulmonary persistent hypertension new-born
PTV: Pressure triggered ventilation
R&I: Research and innovation
REC: Research Ethics Committee
RSS: Respiratory severity score
SAE: Serious adverse event
SpO2: Oxygen saturation
VALI: Ventilator-associated lung injury
VSTT: Ventilatory support tolerance trial
