# Correction: Study protocol for a randomised cross-over trial of Neurally adjusted ventilatory Assist for Neonates with Congenital diaphragmatic hernias: the NAN-C study

**DOI:** 10.1186/s13063-024-07958-5

**Published:** 2024-02-07

**Authors:** Grace Poole, Christopher Harris, Sandeep Shetty, Theodore Dassios, Allan Jenkinson, Anne Greenough

**Affiliations:** 1 Neonatal Intensive Care Unit, King’s College Hospital NHS Foundation Trust, London, UK; 2 Neonatal Intensive Care Unit, St. George’s University NHS Foundation Trust, London, UK

**Correction: Trials (2024) 25, 72**

**https://doi.org/10.1186/s13063-023-07874-0**

Following publication of the original article [1], we have been notified that the 5th author name has been incorrectly spelled as Jenkins instead of Jenkinson. Their affiliation was also incorrectly assigned to no 3 (Neonatal Intensive Care Unit, Kings College Hospital, London, UK) instead of no 1 (Neonatal Intensive Care Unit, King’s College Hospital NHS Foundation Trust, London, UK).

The original article has been corrected and affiliation 3 was removed altogether as redundant.

## References

[1] Poole G (2024). Study protocol for a randomised cross-over trial of Neurally adjusted ventilatory Assist for Neonates with Congenital diaphragmatic hernias: the NAN-C study. Trials.

